# “You have to know why you're doing this”: a mixed methods study of the benefits and burdens of self-tracking in Parkinson's disease

**DOI:** 10.1186/s12911-019-0896-7

**Published:** 2019-08-30

**Authors:** Sara Riggare, Therese Scott Duncan, Helena Hvitfeldt, Maria Hägglund

**Affiliations:** 1 LIME, Health Informatics Centre, Karolinska Institutet, 171 77 Stockholm, Sweden; 2 Karolinska Institutet, LIME, Medical Management Centre, 171 77 Stockholm, Sweden; 3 Norrtälje Hospital, FoUU, 761 29 Norrtälje, Sweden; 4 Department of Women’s and Children’s Health, Uppsala University, 752 37 Uppsala, Sweden

**Keywords:** Parkinson’s disease, Active patients, Self-tracking, PDSA

## Abstract

**Background:**

This study explores opinions and experiences of people with Parkinson’s disease (PwP) in Sweden of using self-tracking. Parkinson’s disease (PD) is a neurodegenerative condition entailing varied and changing symptoms and side effects that can be a challenge to manage optimally. Patients’ self-tracking has demonstrated potential in other diseases, but we know little about PD self-tracking. The aim of this study was therefore to explore the opinions and experiences of PwP in Sweden of using self-tracking for PD.

**Method:**

A mixed methods approach was used, combining qualitative data from seven interviews with quantitative data from a survey to formulate a model for self-tracking in PD. In total 280 PwP responded to the survey, 64% (*n* = 180) of which had experience from self-tracking.

**Result:**

We propose a model for self-tracking in PD which share distinctive characteristics with the Plan-Do-Study-Act (PDSA) cycle for healthcare improvement. PwP think that tracking takes a lot of work and the right individual balance between burdens and benefits needs to be found. Some strategies have here been identified; to focus on positive aspects rather than negative, to find better solutions for their selfcare, and to increase the benefits through improved tools and increased use of self-tracking results in the dialogue with healthcare.

**Conclusion:**

The main identified benefits are that self-tracking gives PwP a deeper understanding of their own specific manifestations of PD and contributes to a more effective decision making regarding their own selfcare. The process of self-tracking also enables PwP to be more active in communicating with healthcare. Tracking takes a lot of work and there is a need to find the right balance between burdens and benefits.

**Electronic supplementary material:**

The online version of this article (10.1186/s12911-019-0896-7) contains supplementary material, which is available to authorized users.

## Background

Parkinson’s disease (PD) is a neurodegenerative condition associated with a wide variety of motor and non-motor symptoms. The manifestations of the disease as well as treatment regimens are often highly individual in nature, making the condition a challenge to manage optimally [1]. PD is primarily diagnosed on the basis of the four cardinal symptoms: tremor, bradykinesia, rigidity and problems with balance and gait. In addition, there are a number of potential other symptoms and medication side effects, related to motor and non-motor functions and the condition can significantly affect quality of life [1, 2]. Median age of onset is 60 years and prevalence increases with age, the incidence between 70 and 79 years of age is 93.1 per 100,000 person years [2]. Medical treatment for PD relies primarily upon oral medications, often with a complicated regimen to follow along with the added risk of side effects. Types, amounts, and combinations of medication prescribed varies with national prescribing patterns [3], age of onset and duration of disease [4], and use of brain surgery [5], but typically the number of tablets increases with duration of disease. A survey study of people with Parkinson's disease (PwP) in the US, France, Germany, Italy, Spain, and the UK found a mean daily intake of about 3 tablets for early stage and about 9 tablets for PwP with advanced stage disease [6]. In advanced disease, brain surgery, such as Deep Brain Stimulation (DBS) and infusion therapy, such as duodopa and apomorhine are also used [1]. The effects of PD as well as the medical treatment are often of a very fluctuating nature and symptoms can differ from one minute to the next [1, 2]. In a previous survey among PwP performed in Sweden, a majority of respondents saw their neurologist for one hour per year or less [7].

Across diseases, patients’ self-tracking of symptoms is an area of increasing interest in healthcare and research. For many diseases, self-tracking of biomarkers and symptoms have been demonstrated to improve disease management [8] and clinical outcomes [9].

Self-tracking can be supported by the use of technology and different technical solutions for clinical use is an area of substantial interest in PD. The views of clinicians of the uses of technology seem to differ somewhat from that of PwP. Clinicians expressed that technology is most valuable when it can be related to an effective therapy [10], meaning mainly motor symptoms. Furthermore, that the main reasons for using technology are 1) research and 2) evidence-based medicine and clinical care [11]. PwP consider motor and non-motor symptoms equally important [12] and expect technology to be able to capture the full complexity of PD [13]. A Swedish focus group study of the potential benefits and barriers of using wearable technology for PD and epilepsy found that PwP saw a potential to use technology for their own self-management including adjustment of medication [14]. Healthcare professionals in the same study expressed concerns about PwP adjusting their medication based on tools not provided from the clinic.

The temporal variability of PD symptoms in combination with the limited time PwP have with their clinicians implies a substantial potential for improvement with the use of self-tracking to achieve a better understanding of the condition. Previous studies have focused on clinical aspects [15] and hypothetical discussions [14]. To the best of our knowledge, the only previous study investigating the practical patient perspective on self-tracking in PD is a case study by two of the four authors to this study [16]. No studies have been reported looking at a larger group of PwP and their self-tracking practices, to see if there is a potential for a better understanding regarding their condition. What contribution can access to more objective data about one’s own symptoms and healthmake? The aim of this study was therefore to explore the opinions and experiences of PwP in Sweden of using self-tracking for PD with a focus on variety of experiences rather than representativity.

## Method

To identify the opinions and experiences of PwP regarding self-tracking, a mixed method approach and triangulation were used, to combine qualitative data from interviews with quantitative data from a survey. A mixed methods research approach can be suitable when aiming to explore a research question in both breadth and depth [17].

### Study design

**Fig. 1:**
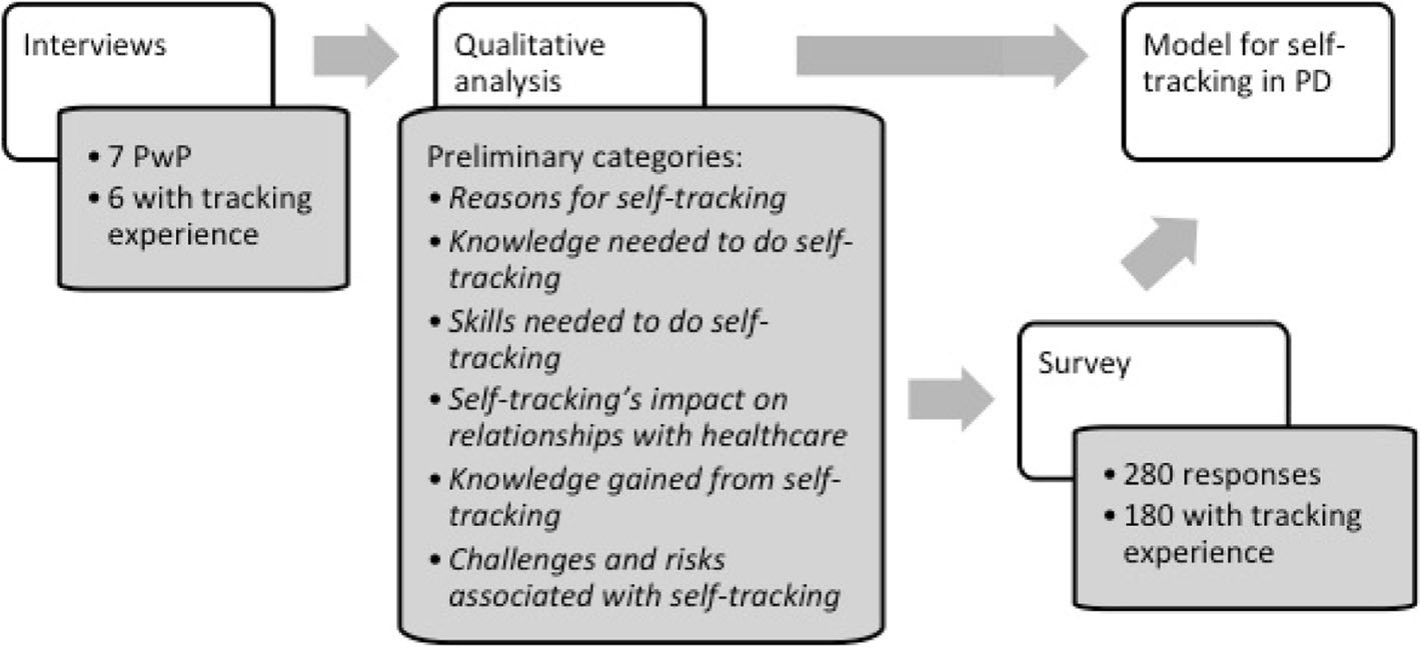
Study design

We chose to combine data from interviews with results from a survey. To increase our understanding for self-tracking in the context of PD, we first conducted in-depth interviews with PwP (*n* = 7) with experience of performing self-tracking. The results of these interviews then informed the design of a survey distributed more widely in the Swedish PD community. Analysis of both interviews and survey results informed the design of a model for self-tracking in PD. An overview of the study design is presented in Fig. 1.

### Qualitative data collection

It was a purposive sampling [18] by one of the authors (SR), recruiting participants from personal networks and contacts from previous work. We wanted to elicit the views and opinions of PwP on self-tracking and were looking specifically for PwP with personal experience of self-tracking. Our respondents had all expressed an interest in self-tracking and all except one had personal experience of self-tracking. In order to obtain a broad perspective, efforts were made to find participants with varying backgrounds, ages, geographic location, and PD characteristics (current treatment, severity of disease). The participants all have PD without cognitive decline. Table 1 gives an overview of the participants.

**Table 1:** Characteristics of interview respondents (*n* = 7)

Gender (men/women), *n*	3/4
Age (years), median (min/max)	58 (52/67)
Years since diagnosis, median (min/max)	5 (3/13)
Current PD treatment, (oral/advanced)	(7/2*)
Personal experience of self-tracking, *n* (N)	6 (7)

*One DBS, one Duodopa

A semi-structured interview guide was developed specific for this study, containing questions related to information on background, disease characteristics (time of diagnosis, symptoms), interactions with healthcare relating to PD, and self-tracking (see interview guide as Additional file 1). The interview guide was pilot tested with three PwP and minor adjustments were made prior to the first interview. Interviews with other PwP than the ones from the pilot test were conducted between October 2015 and August 2016. The duration of all the interviews was 283 min.

The first author, who is part of the PD community, conducted the interviews using an iterative approach with the help of another PwP. After each two-three interviews, a preliminary analysis was performed to evaluate the level of saturation [19]. The interviews were continued until saturation was reached related to the topic of self-tracking.

During the interviews, background information (age, time since diagnosis, current treatment etc) was collected see Table 1.

### Qualitative analysis

The interviews were recorded, transcribed verbatim and analysed qualitatively using inductive qualitative content analysis [20, 21]. The analysis was conducted in Swedish in order to stay as true as possible to the meaning of the text [22]. Two of the authors (SR and TSD) listened to the recordings, read the transcripts and sorted the text into two content areas: self-tracking and collecting data in collaboration with healthcare. SR and TSD then independently selected the relevant data into one text (the unit of analysis). The data selection was verified among all four authors. In the next phase, the text was divided into condensed meaning units that were abstracted and labelled with codes. The codes as a whole were compared and organised in preliminary sub-categories and categories, representing the manifest content of the data. All four authors met several times to compare and discuss the codes, sub-categories, and categories. Where opinions varied, the cases were discussed until consensus was achieved. In the final phase of the analysis, in order to increase credibility, illustrative quotes were selected and translated to exemplify each category. The translations were verified by all authors.

The qualitative analysis of the interviews resulted in six distinct categories, namely Reasons for self-tracking, Knowledge needed to do self-tracking, Skills needed to do self-tracking, Self-tracking’s impact on relationships with healthcare, Knowledge gained from self-tracking, and Challenges and risks associated with self-tracking (see also Fig. 1). These preliminary categories were used to design the survey.

### Quantitative data collection

Based on the categories from the qualitative analysis of the interviews, a survey was developed in Swedish, specific for this study (see the survey as Additional file 2). Since 91% of the Swedish population uses the internet [23], an online survey was considered appropriate. The survey was designed and distributed using Google Forms. General information about the survey (purpose of the study, investigator, and instructions for responding) was included. The survey comprised six sections; background, experience of self-tracking, reasons for self-tracking, approach and use of self-tracking, self-tracking’s influence on relationships with healthcare, and challenges and risks associated with self-tracking. Responses included multiple-choice options (with both “check only one” and “check all that apply”) and a five-tiered Likert scale (with options *strongly disagree*, *disagree somewhat*, *neither agree nor disagree*, *agree somewhat*, and *strongly agree)*. Questions were kept short and focussed to reduce the risk of respondents abandoning the survey before completion [24].

The link to the survey was distributed via patient organisations, social media and personal networks. Since our focus was on variety rather than representativity, we prioritised a wide reach over being able to calculate response rate. Data were collected between December 7, 2017 and January 7, 2018.

### Quantitative analysis

For the purpose of analysis, the five-tiered rating was replaced by the options *disagree* (including the options *strongly disagree* and *disagree somewhat*)*, neutral, agree* (including the options *strongly agree* and *agree somewhat)*. An online calculation tool [25] was used for statistical analyses using the χ2 test with statistical significance defined at *p* < .05.

## Results

The results from the interviews and the survey were combined to create a conceptual model describing our understanding of self-tracking in PD. Analysis of the qualitative and quantitative data resulted in five distinctive categories, which are further described in the following sections. The categories are: *Why I self-track*, *How and what I self-track, Lessons learned from self-tracking, Risks related to self-tracking,* and *Self-tracking and healthcare*.

### Background data

In total, the survey had 280 complete responses from Swedish PwP whereof 180 had experience from self-tracking. The characteristics of the respondents, including age, time since diagnosis and education level are given in Table 2 (with self-tracking experience).

**Table 2:** Characteristics of survey respondents with self-tracking experience (*n* = 180)

Age	**26–35 yrs**	**36–45 yrs**	**46–55 yrs**	**56–65 yrs**	**66–75 yrs**	**76–85 yrs**	**> 86 yrs**
Women	1	6	14	28	30	9	
Men			11	23	46	11	1
%	1%	3%	14%	28%	42%	11%	1%
Time since diagnosis		**< 1 yr**	**1–5 yrs**	**6–10 yrs**	**11–15 yrs**	**16–20 yrs**	**> 20 yrs**
Women		5	38	25	17	2	1
Men		1	31	32	17	8	3
%		3%	38%	32%	19%	6%	2%
Highest completed education level		**Compulsory school (< 9 yrs)**		**Upper secondary school (9–12 yrs)**		**University (> 12 yrs)**	
Women		5		28		55	
Men		10		25		57	
%		8%		30%		62%	

The entries with bold text in the table are to show the different characteristics of the survey respondents; age, time since diagnosis and highest completed education level, in comparison to gender

**Fig. 2:**
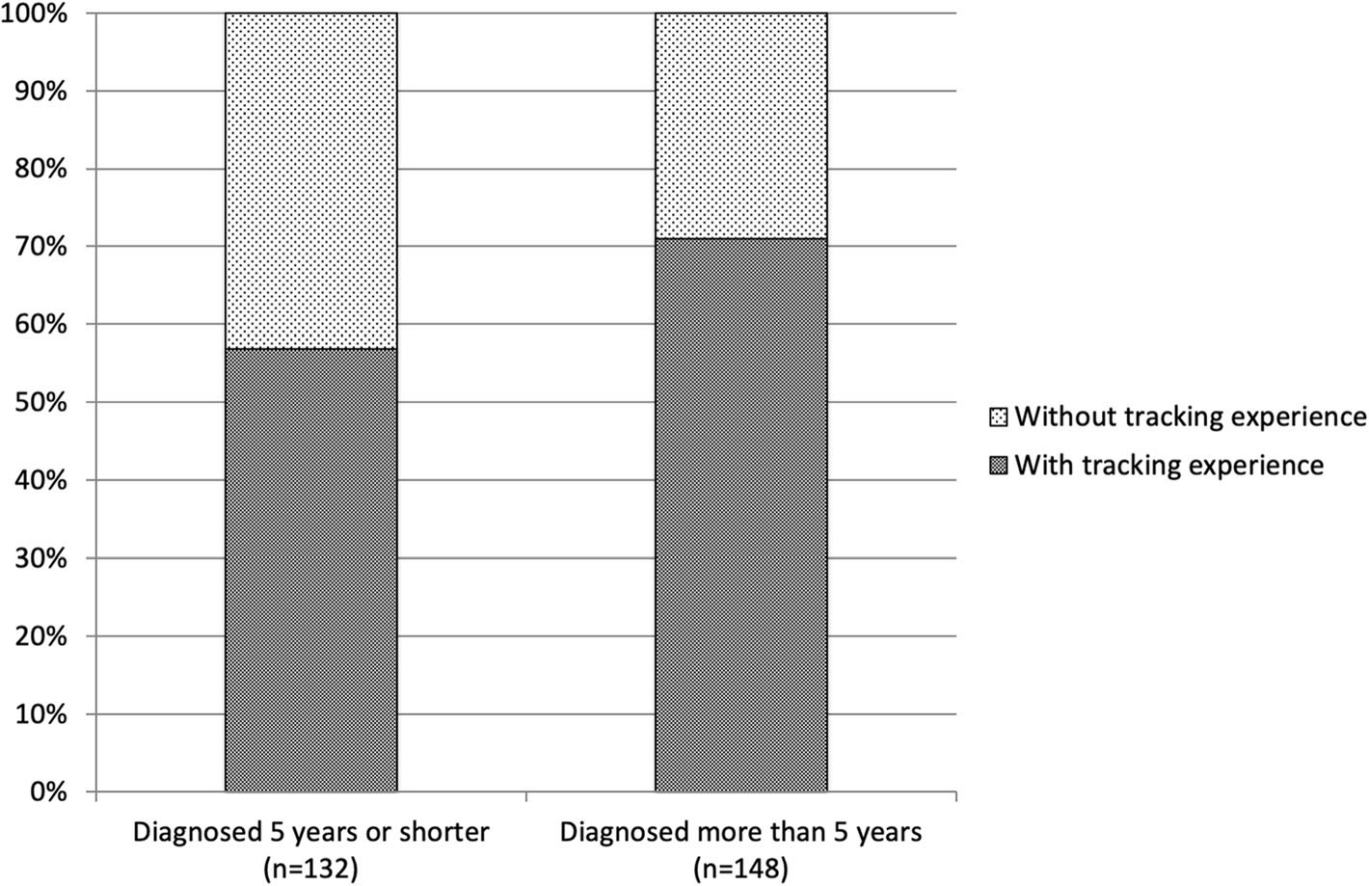
Self-tracking experience for PwP diagnosed for different lengths of time

Regardless of self-tracking experience or not, the respondents were similar in age distribution, education levels and proportion male/female. There was however, a significant difference regarding time since diagnosis (see Fig. 2); survey respondents who had been diagnosed more than five years were more likely to have tried self-tracking (71%) than PwP diagnosed five years or shorter (57% have tried self-tracking) (χ2(2) = 6.066, *p* = .014).

To meet the aim of the study, the analyses and construction of the model were made using the survey results from PwP with self-tracking experience (*n* = 180). Of those respondents 49% were women, average age was 64.4 years and average time since diagnosis was 7.7 years.

### Why I self-track

All respondents in the interviews talked about reasons for self-tracking. Several of them mentioned that they have a mind-set for self-tracking and therefore may be more interested than PwP in general. They also demonstrated an awareness of the highly individual presentation of PD, that the illness will be very different for different individuals and that each person needs to understand their own symptoms and how to manage them. Some respondents also described self-tracking as a means to achieve increased awareness of their illness and its progression.

*R3: “I expect tracking to help me more clearly see how my disease really is, now it’s mostly guesswork.”*

**Fig. 3:**
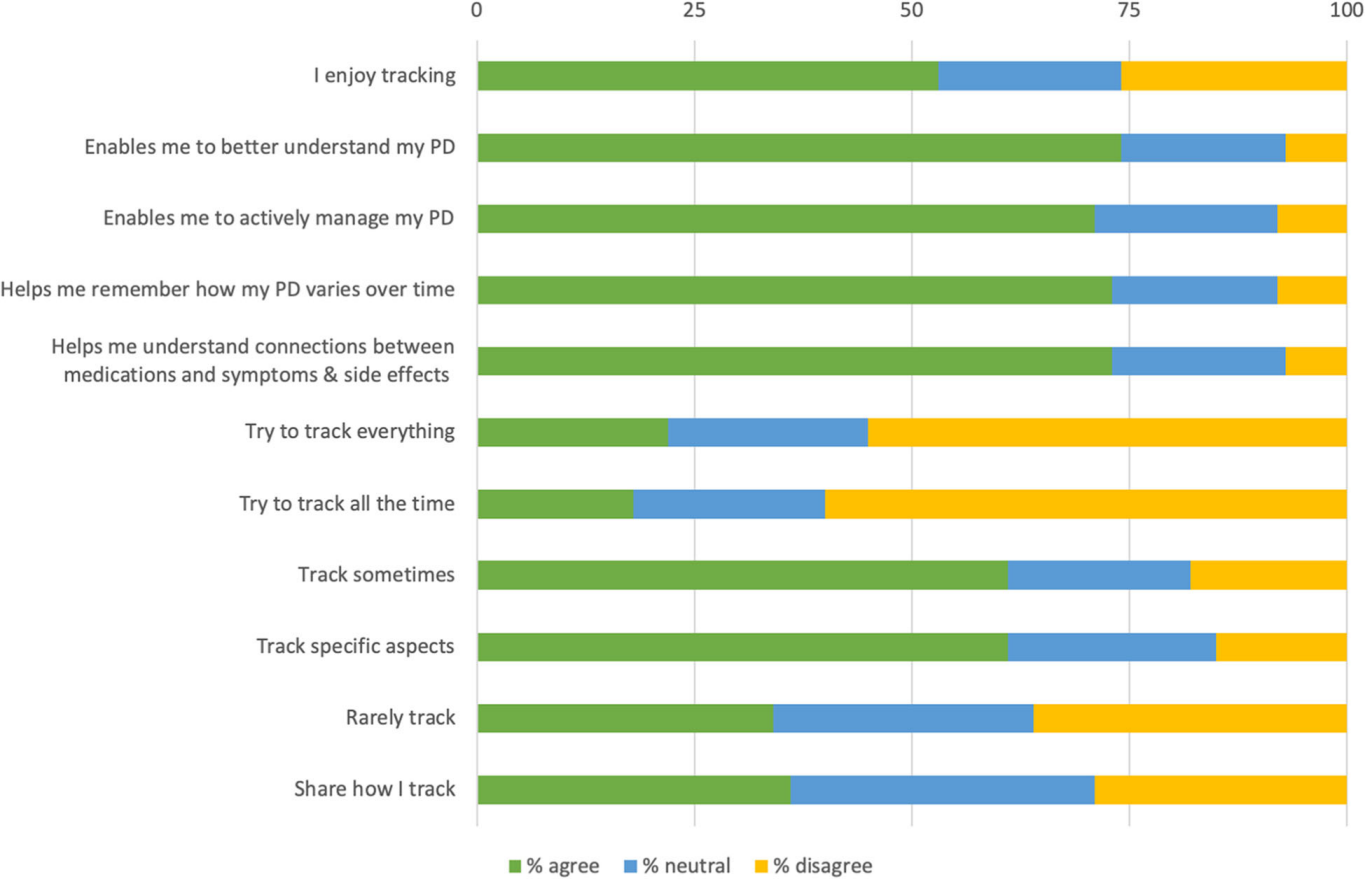
Why I self-track

Similarly, in the survey (see Fig. 3), the most common response to why people self-track, was that they expect it to enable them to understand their PD better (74%).

**Fig. 4:**
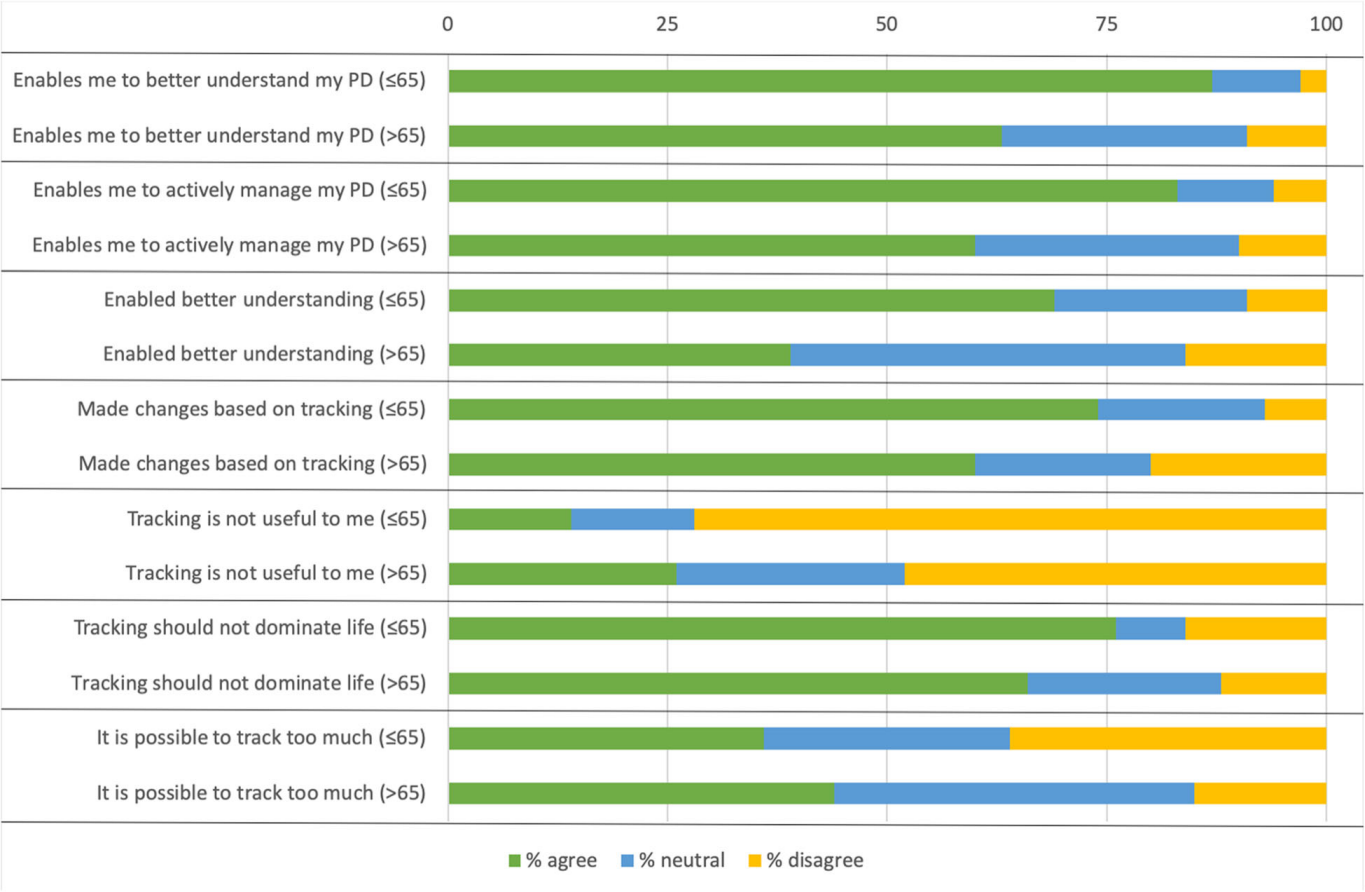
Significant differences between PwP younger and older than 65

PwP younger than 65 are significantly more likely to state that self-tracking enables them to understand their PD better (87%) than PwP older than 65 (63%) (χ2(2) =13.215, *p* = .001), see Fig. 4.

The interview respondents also mentioned self-tracking as a tool to find and understand correlations between health status or symptoms and medication intakes. It is a way to recall how they were doing over time, for example to remember how symptoms fluctuate. Both of these reasons were chosen by 73% of the respondents of the survey, see Fig. 3.

Some of the interview respondents talked about how they take an active approach in the management of their PD. Self-tracking was described as a way to stay in control, and to take personal responsibility for one’s health using an active approach. In the survey, 71% stated that tracking enabled them to take an active approach in the management of their PD, see Fig. 3. PwP younger than 65 were also significantly more likely to take an active approach (83%) than PwP older than 65 (60%) (χ2(2) =12.13, *p* = .002) see Fig. 4.

*R2: “To me, it’s positive that it makes me more aware. You can’t stick your head in the sand, the disease will catch up with you no matter what you do.”*

**Fig. 5:**
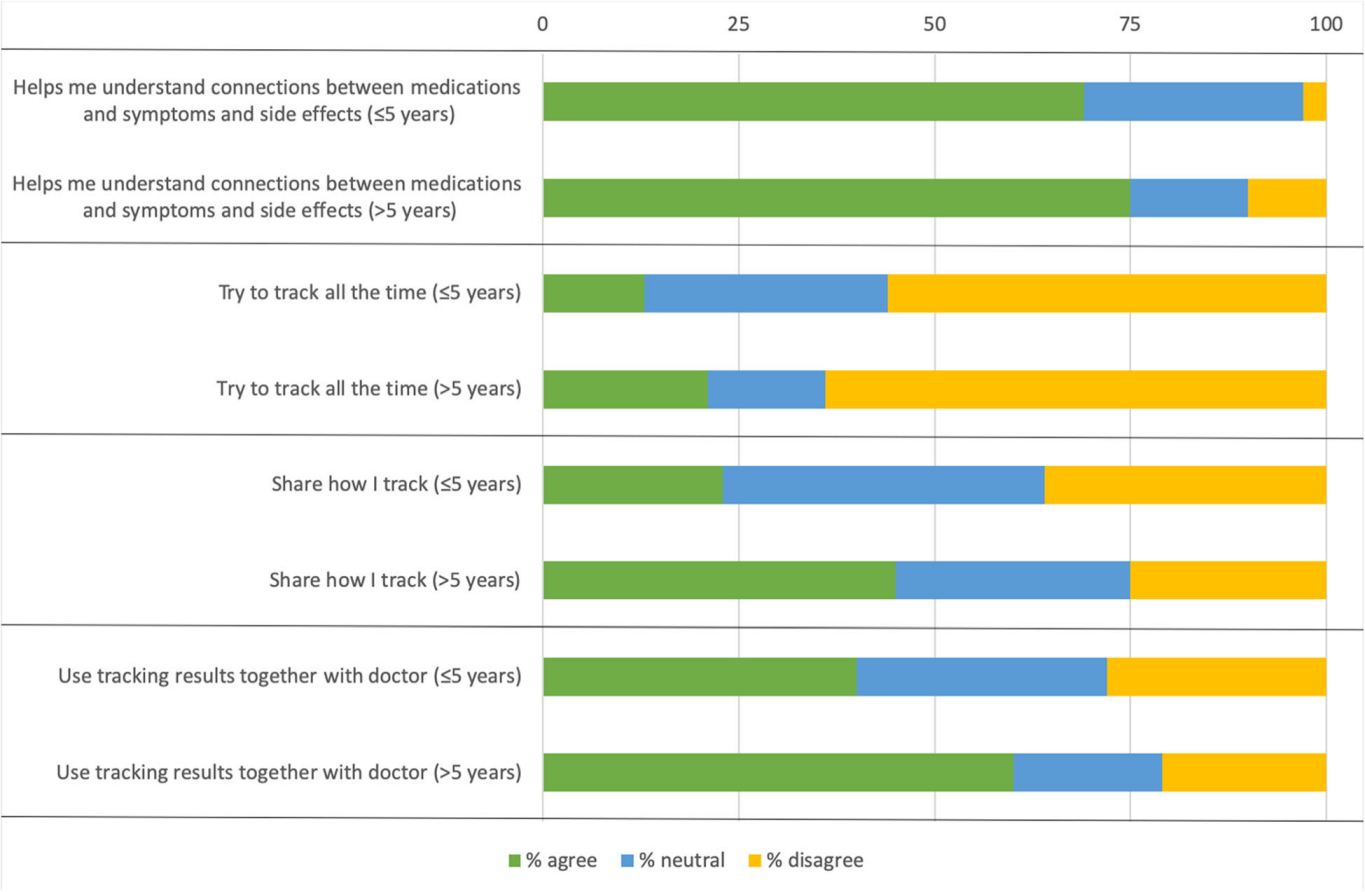
Significant differences between PwP diagnosed less or more than five years ago

In Fig. 3 is also presented that about one in two of our survey respondents (53%) state that they self-track because they enjoy it. However, they also think it is challenging to track; 58% find it difficult to know what to track and 61% find it difficult to know how to track. No more than 22% say that they try to track everything and 18% say they try to track all the time. In contrast, 61% say they track sometimes and that they track specific aspects of their PD. PwP who have been diagnosed for more than five years are significantly more likely to try to track all the time (21%) than those who were diagnosed less than five years ago (13%) (χ2(2) =6.676, *p* = .04), see Fig. 5. In total 36% of PwP share their experiences of tracking with others, see Fig. 3. Those who have been diagnosed for more than five years are significantly more likely to share with others (46%) compared to those diagnosed less than five years ago (23%) (χ2(2) =10.084, *p* = .006), see Fig. 5.

### How and what I self-track

Our interview respondents described knowing what and how to track as important. The primary parameter to track among the participants was medication intakes, with the purpose to optimise timings. Other parameters important to measure were stress, diet and sleep. Different influencers were also acknowledged as a challenge in the interpretation of the collected data, and several participants described how important it was to understand these complexities in order to benefit from tracking.

*R1: “It’s important to take your medication right, at the right time. You can get a bad effect, it doesn’t always mean that you need to increase your dose, it can mean that you need to make it more evenly distributed.”*

The respondent with the most experience of tracking also expressed more abstract reasoning around how to capture the right measure when tracking, and what effects the choice of measurement can have. The same respondent also reflected on the challenges of defining more subjective measures to find a measure that is both efficient and reliable. What does it mean to me to “feel well”?

*R1: “It was really hard work to constantly think about whether I didn’t feel pain somewhere, if I didn’t feel stiff and so on… It took over my life... So I realised that I have to register something else and I decided to make notes of when I am doing well instead, when my symptoms are on the level I want them to be.”*

Tools used for measuring include different kinds of activity trackers, smartphone apps for tracking sleep, exercise or similar. Several of the respondents used spreadsheet programs (e.g. Microsoft Excel) for gathering data and making graphs or other visual representations of the data.

From the 180 included survey respondents, 49% had used some kind of technology, for their tracking. It could be a computer, smartphone, tablet, sensors or other devices, like smart watches, see Table 3. More frequently used was pen and paper (56%), and 74% had kept track in their head. Mode of tracking seems to be unrelated to gender, age, and education level. When it comes to time since diagnosis however, PwP diagnosed more than five years ago were significantly more likely to track using pen and paper (66%) than those diagnosed five years ago or less (41%) (χ2(1) =10.533, *p* = .001), see Table 3.

**Table 3:** Mode of tracking

I have experience of self-tracking using:	Technology		Pen and paper		My head	
	% agree	% disagree	% agree	% disagree	% agree	% disagree
Dx 5 years ago or less	44	56	41	59	75	25
Dx more than 5 years ago	53	47	66	34	74	26
Grand total	49	51	56	44	74	26

Diagnosed 5 years ago or less, diagnosed more than five years ago, and grand total

**Fig. 6:**
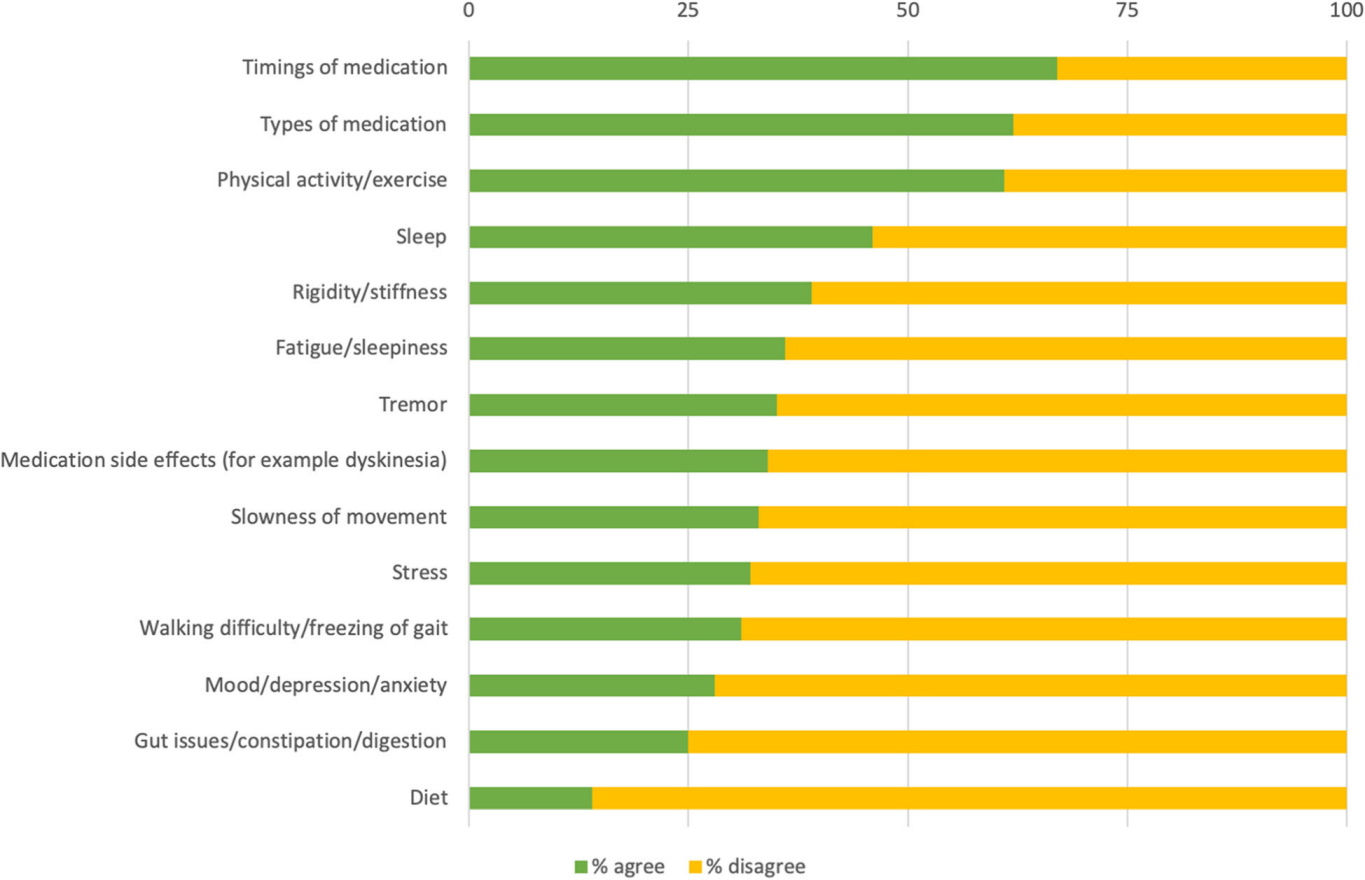
Aspects of PD tracked

The most common aspects to track in the survey were medication intake times (67%), medication types (62%), and physical activity/exercise (61%), see Fig. 6.

### Lessons learned from self-tracking

Another category emerging from the data was *lessons learned from self-tracking*. The category includes insights gained from exploring tracking as well as barriers and challenges.

Several of the interview respondents mentioned that they had to learn a lot themselves to develop the understanding for the causalities and inferences necessary to really benefit from tracking.

*R5: “I have gathered a lot of knowledge for myself in order to understand the connections. I found knowledge by reading, online, patient associations, conferences.”*

They had gained interesting insights and concrete results from their tracking and described how they had realised what was important for understanding their PD. For example how their medication was connected to sleeping patterns or a desire for sweets and how their ability to engage in physical activity varied over time. The respondents used the knowledge gained from tracking in different ways: for example for tweaking their medication regimen or for adjusting their food intake.

*R6: “It’s difficult to tweak medication timings, there are so many influencing factors; stress, food, lack of sleep, it’s all inter-connected.”*

**Fig. 7:**
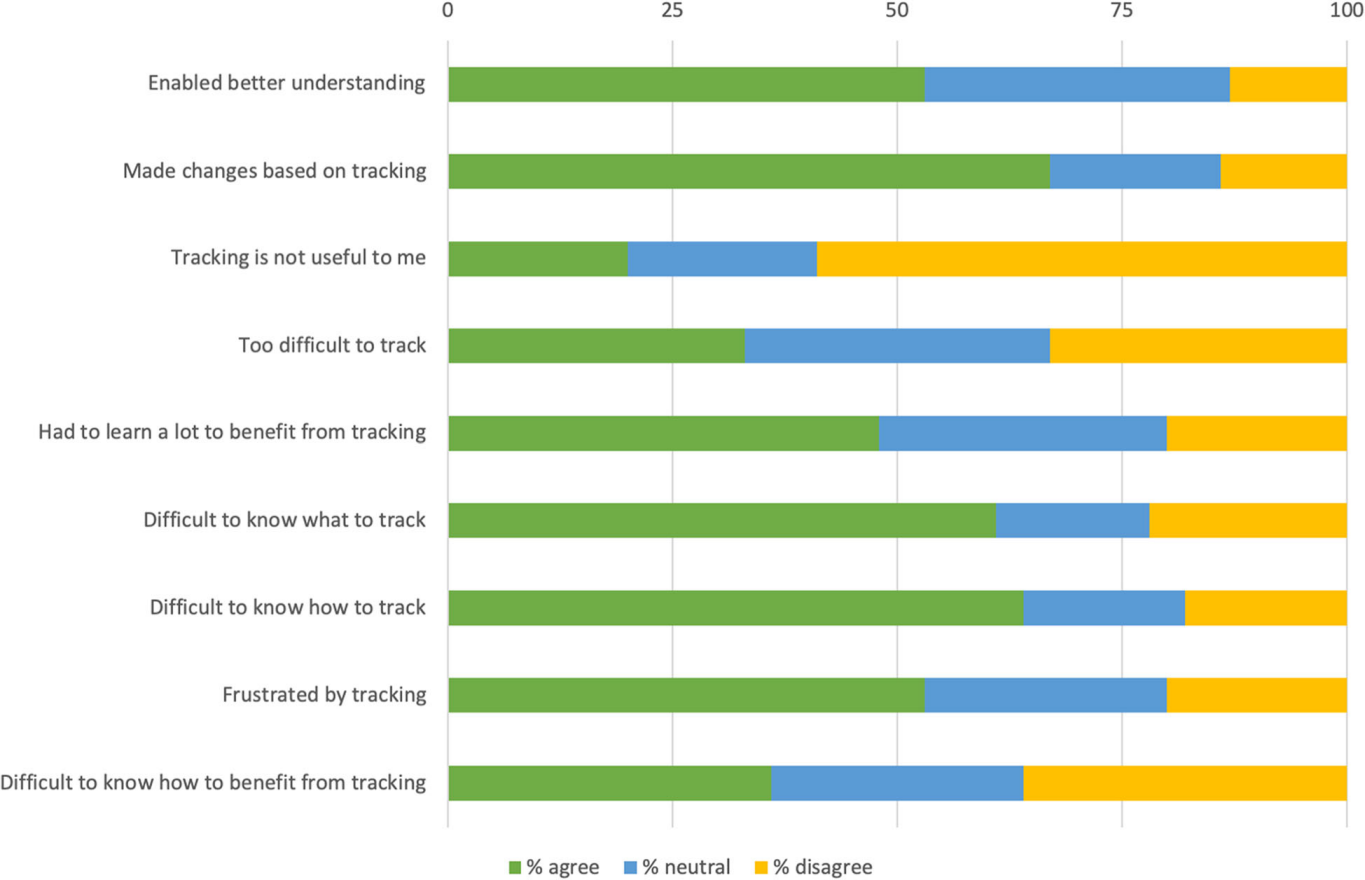
Lessons learned from self-tracking

In the survey results, the ways the respondents utilise tracking varies: 51% said that they have made observations through their tracking that has enabled them to understand their PD better and 67%, said that they have made changes to their PD management as a result of tracking, see Fig. 7. For PwP under 65, this rate was significantly higher (69 and 73% respectively) compared to those older than 65 (39 and 61% respectively) (χ2(2) =15.841, *p* = .0004; χ2(2) =5.998, *p* = .05), see Fig. 4. One in two, 53%, were frustrated with how difficult it is to track and 36% think it is difficult to know how to make use of the results of tracking, see Fig. 7.

### Risks related to self-tracking

Potential risks related to tracking are the fixation of tracking and not to let it take over your life. Most frequently mentioned by our interview respondents was the risk of PwP getting fixated or obsessed with tracking. This can be related to effects of the disease itself and/or medication. The importance of finding a balance in life and not let the tracking get in the way of living was also mentioned. The respondents stressed that there has to be a balance between tracking to learn about your own condition and giving the disease too much focus.

*R1: “I don’t think you should be doing it all the time if you don’t know what you want to use it for. Just tracking, that’s pointless. You have to know why you’re doing this.”*

**Fig. 8:**
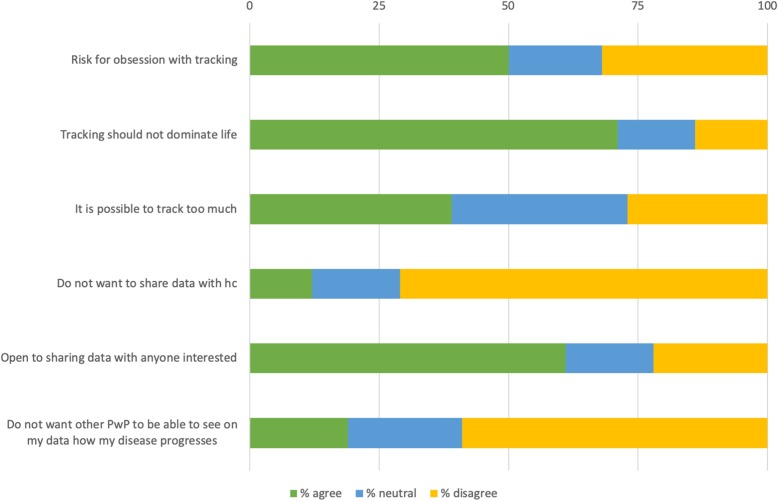
Risks related to self-tracking

Our included survey respondents also see risks associated with tracking; one in two, 51%, think there is a risk of becoming obsessed with tracking PD, see Fig. 8. A major part of respondents, 71%, do not want tracking to become too large a part of life (see Fig. 8) and PwP younger than 65 are significantly more concerned (76%) than those older than 65 (66%) (χ2(2) =5.995, *p* = .05), see Fig. 4. In total 39% believe that it is possible to track too much (see Fig. 8) and PwP older than 65 were more likely to agree with that statement (44%) than those younger than 65 (36%) (χ2(2) =10.677, *p* = .005), see Fig. 4.

On the issue of data privacy, our interview respondents in general expressed a trust in the healthcare system and did not see any major risks when it comes to sharing their data, neither with healthcare nor with other patients or caregivers. Sharing was considered positive as long as it was on their terms and with appropriate security measures in place. Our survey respondents were not reluctant to sharing data from their tracking; 72% were positive to sharing with healthcare and 61% were open to sharing with anyone who is interested, see Fig. 8.

### Self-tracking and healthcare

The practise of tracking can influence the relationships with healthcare, both in a positive and a negative way and this is described in the category *self-tracking and healthcare*.

Some of our interview respondents used tracking results as a trigger to contact healthcare to handle worsening symptoms. They expected healthcare to be interested in tracking data, since this kind of information is difficult for healthcare to come by without patients collecting the data themselves. One expected benefit was that it would save time at the doctor’s visit if PwP collected data beforehand. Tracking as memory support was considered important in preparation for clinical encounters and to give their clinicians an accurate account of their disease status since the previous appointment.

*R7: “Sometimes I have been allowed to present my tracking but there doesn’t seem to be much interest from healthcare. I think it has to do with the attitudes of doctors, I get the feeling that they want to do their assessments without involving my tracking.”*

**Fig. 9:**
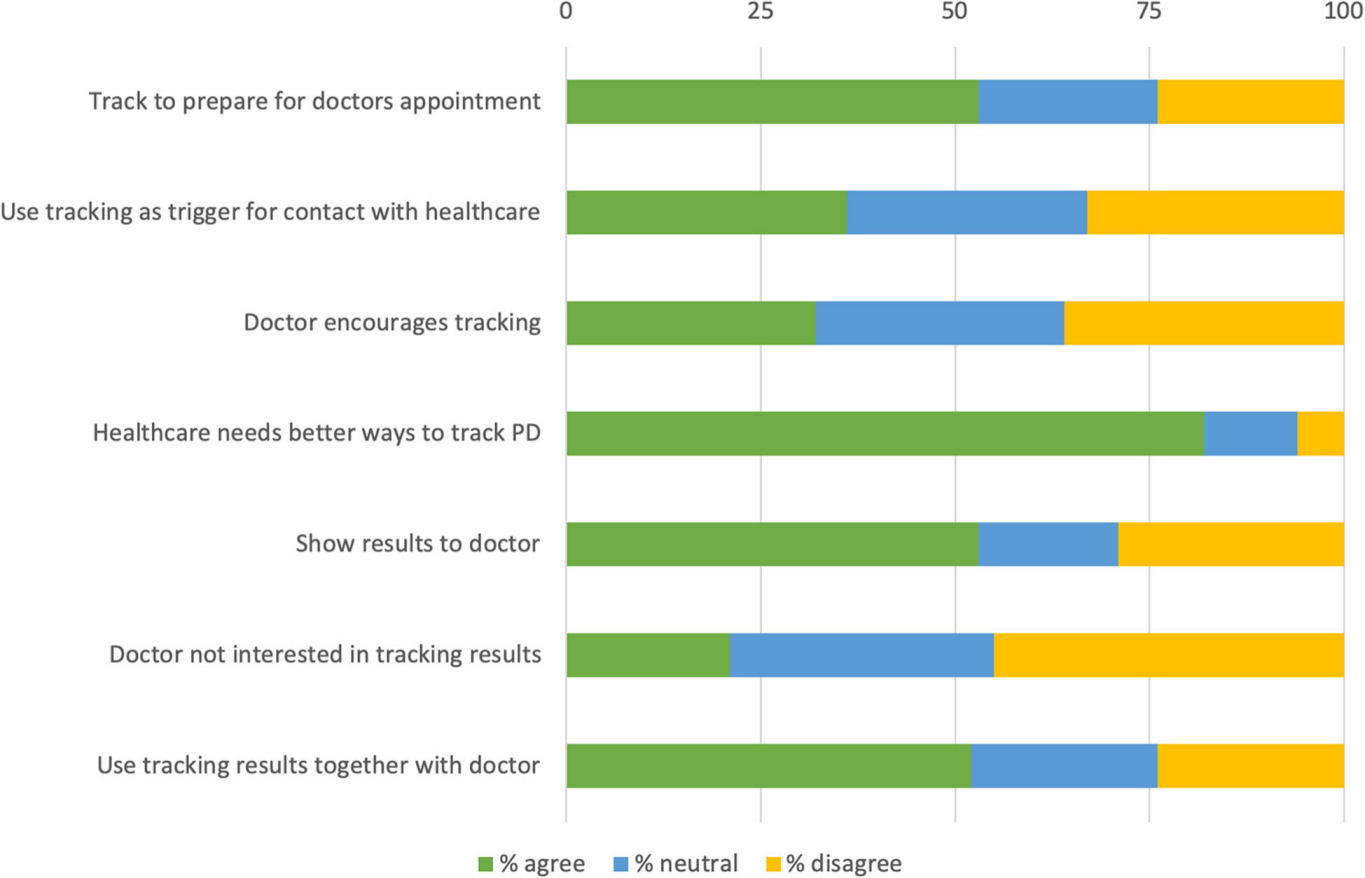
Self-tracking and healthcare

In total 36% of our 180 included survey respondents used tracking to decide if they needed to visit their physician and 53% said that they track to prepare for healthcare visits, see Fig. 9.

**Fig. 10:**
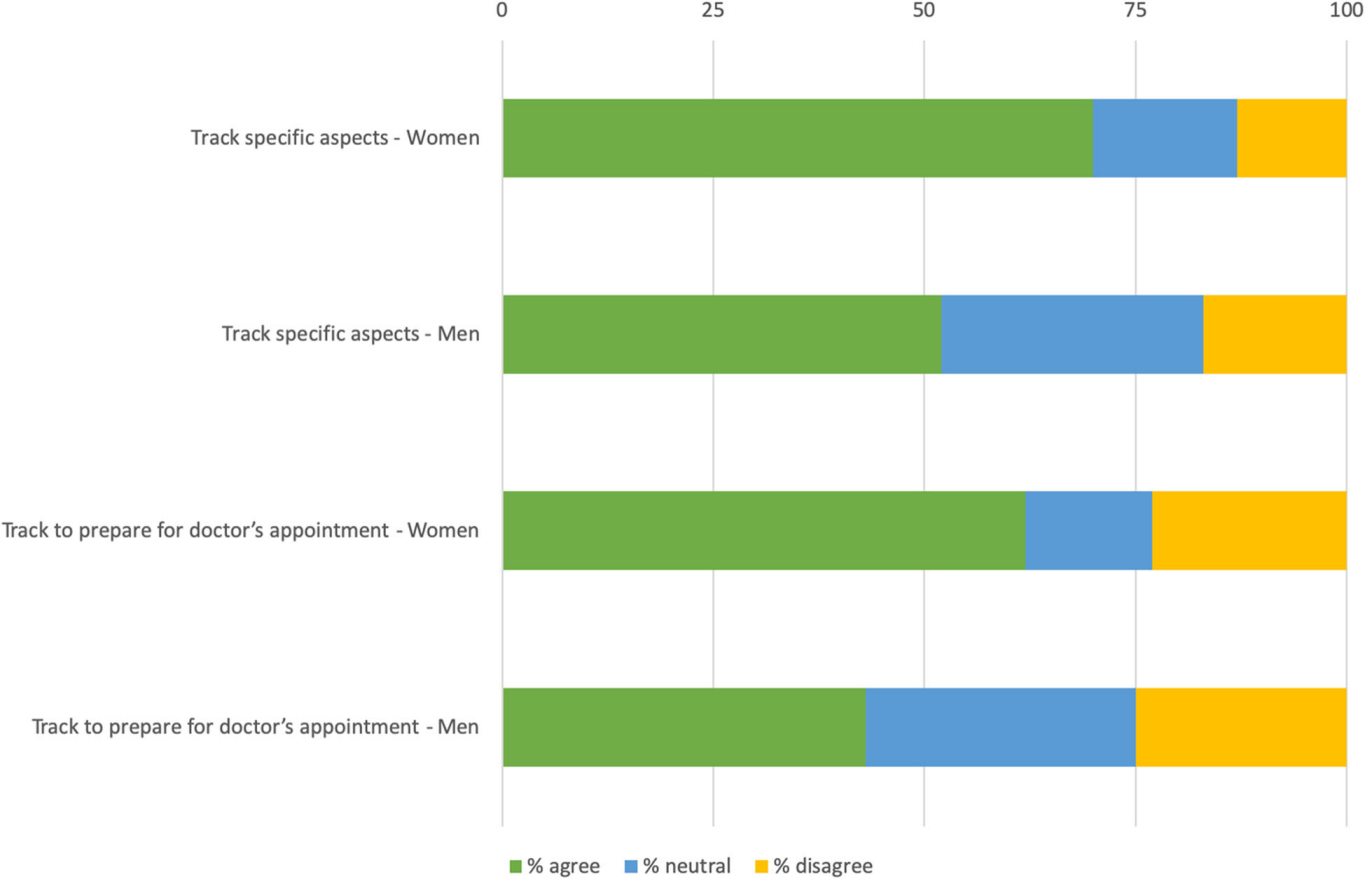
Significant differences between women and men

Women are significantly more likely to track to prepare for healthcare visits than men (63% compared to 43%) (χ2(2) =8.588, *p* = .02), see Fig. 10.

The interview respondents expressed a wish to collaborate with healthcare around tracking. There can be a potential benefit from making use of data that are collected in-between healthcare encounters. At the same time, our respondents showed understanding for the challenges healthcare professionals face, which can explain healthcare professionals hesitation for encouraging tracking. In general, the respondents appreciated that it can be difficult for healthcare professionals to change the way they are working. Financial concerns were also mentioned, as well as the need for bringing context to the tracking data. It was also expressed that both healthcare and PwP need better tools and support for analysing tracking data. Several of our respondents demonstrated how the tracking they undertook and the knowledge they gained from this enables them to be more active in communicating with healthcare.

*R4: “... and the doctor says…: ‘Let’s replace [medication X] with [medication Y], and you can take it in the evening’. ‘But if I do that, I won’t be able to work because I will have more tremor in the mornings.’ ‘Yes’ she said. ‘But, can’t I take [medication X] in the morning instead?’ I said. ‘And I will be able to work’, I said. And if I hadn’t known this, I would have taken her suggestion and I would have functioned less well in daytime, and maybe I wouldn’t have been able to work as much.”*

Figure 9 shows that more than one in two PwP in the survey (53%) showed their tracking results to their physician. However, only 32% said that their physician encourages them to track and only 21% said that their physicians are interested in their tracking. In total 52% of PwP said that their tracking results are used in the clinical encounter to make treatment decisions and for those diagnosed more than five years ago, the rate was significantly higher (60%) compared to those diagnosed less than five years ago (40%) (χ2(2) =7.299, *p* = .03), see Fig. 5. As many as 82% of our respondents thought that healthcare needs to find better ways to assess PD on an individual level, see Fig. 9.

### Model for self-tracking in PD

**Fig. 11:**
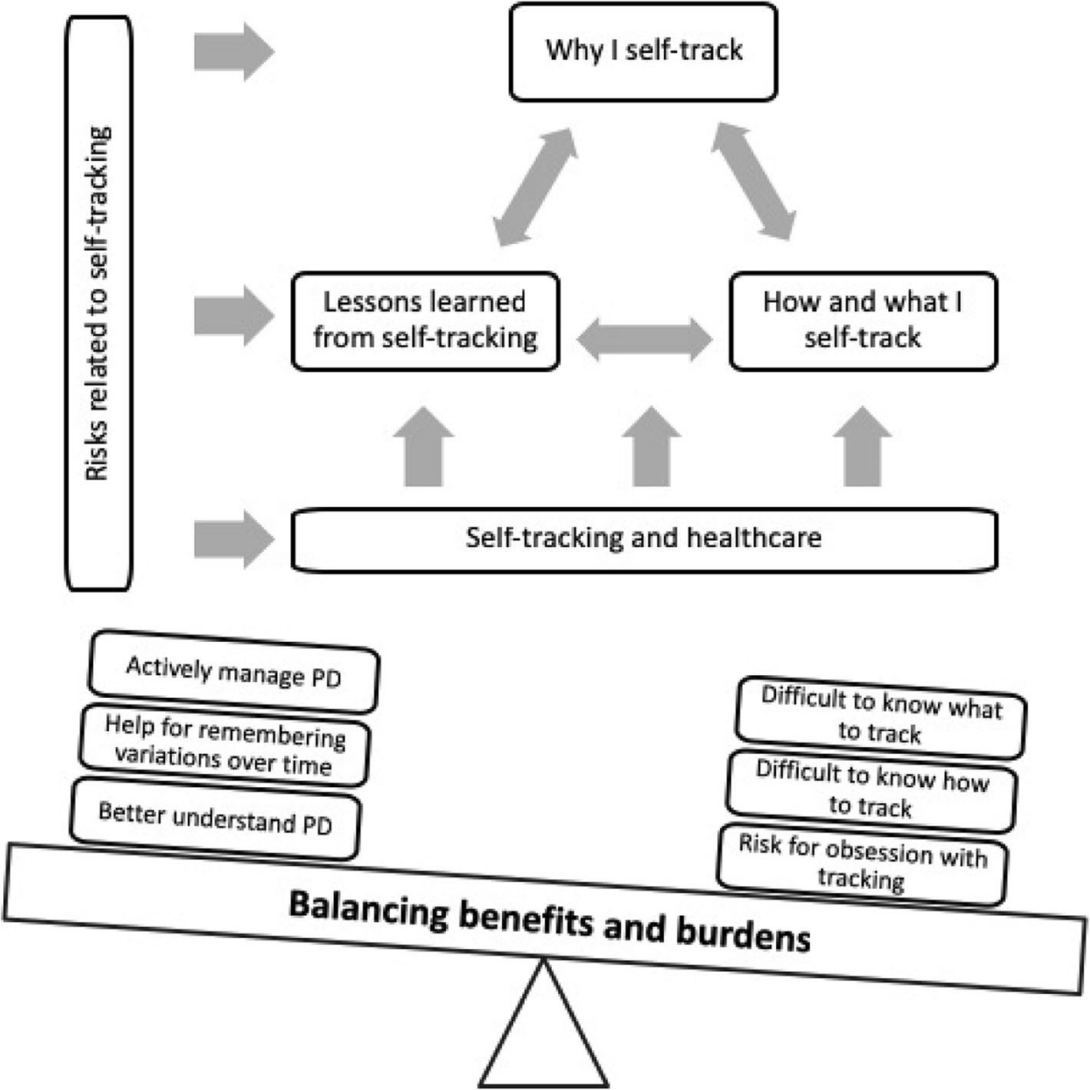
Balancing benefits and burdens

Figure 11 shows the proposed model based on the analysis of our study results. Core concepts in the model relate to the motivation and drive to self-track (*Why I self-track*), skills and tools required (*How and what I self-track*), and the knowledge produced from self-tracking (*Lessons learned from self-tracking*). Risks were mentioned across all these areas, as were the mixed experiences of utilising self-tracking in collaboration and communication with healthcare.

When working with the categories we found that they were connected via a theme, going across the data. The respondents’ descriptions and experiences demonstrated that there is a lot of work associated with self-tracking and that the respondents expressed both benefits and burdens. We found that an overarching theme emerged, namely *Balancing benefits and burdens*. Examples of mentioned benefits and burdens are given in Fig. 11 and some of the strategies for finding this balance that emerged from the interviews will be addressed in the Discussion section.

## Discussion

The aim of this study was to explore the experiences and opinions of people with PD in Sweden on self-tracking and the results presented above indicate that it can be both rewarding and challenging. We found that the “average PwP self-tracker” track different aspects of their disease and treatment, for example timings and/or types of medication, and exercise or physical activity. Mostly they track only in their head, but sometimes they also make notes on paper or use technology. They see tracking as a tool they can use to better understand and actively manage their PD. They track specific aspects occasionally, for example to help them understand how medications relate to symptom relief and side effects and to remember how their disease varies over time. Tracking can be frustrating, it can be difficult to know what and how to track and they strongly believe that healthcare needs better ways to assess PD. They track to prepare for doctor’s appointments and use the tracking results together with their doctor. When it comes to sharing their tracking data they are open to do so with healthcare and other people interested. They do see a risk for becoming obsessed with tracking and think it is important that tracking does not dominate life.

We propose that self-tracking as described by our respondents, could be seen as a personal improvement project, aiming to understand and improve ones own health and disease management. Continuous quality improvement in healthcare is often based on the model for improvement - PDSA cycle - introduced by Edwards Deming and further developed by Nolan et al. [26]. The PDSA cycle approach is an iterative process for learning and improving complex systems and resembles the process of an individual learning about their own health in order to improve it. The approach has in fact also been used in trying to improve individual health in chronic disease [27]. Three fundamental questions form the basis for improvement, according to [26], namely:

1. What am I trying to accomplish?
2. How will I know that a change is an improvement?
3. What changes can I make that will result in improvement?

The PDSA approach resonates well with the categories emerging from our study results, see Fig. 3. We posit that our category “why I self-track” corresponds to the first of the fundamental questions, our category “how and what I self-track” to the second question, and our category “lessons learned from self-tracking” to the third.

The theme of balancing benefits and burdens that we found in our data is not as clearly expressed in the PDSA cycle. It is however present in the notion that it has to be possible to determine whether an improvement has occurred – otherwise the change is potentially only a waste of efforts and resources. When patients engage in personal improvement related to their health the stakes are even higher and require other strategies for finding the balance between benefits and burdens, as we will discuss in more detail below.

Women in our survey are more likely than men to track specific aspects as well as to track in preparation for doctors’ appointments. For PwP younger than 65, there are some significant differences compared to the group as a whole. When it comes to making use of tracking, people in the younger group are more likely to have made changes to for example their treatment as a result of tracking. Younger PwPs are also more likely to disagree with the statement that tracking is not useful for them. Our results also indicate that the older group are more likely to agree that it is possible to track too much. Time since diagnosis seems to be the most important factor when it comes to attitude and use of self-tracking. In total, 64% of our survey respondents have experience from tracking. The characteristics of trackers and non-trackers are not significantly different when it comes to age, gender distribution, or education level. The groups are however different when it comes to time since diagnosis; PwP diagnosed more than five years ago are significantly more likely to self-track than PwP diagnosed for five years or less. Furthermore, PwP diagnosed more than five years ago are significantly more likely to consider tracking helpful, to share their learnings from tracking with others, and to use tracking together with their doctor, than PwP diagnosed a shorter time.

Research on tracking in other conditions and contexts has made findings of relevance to our study. A study exploring tracking in multiple chronic conditions found that patients considered tracking to be “illness work” and that it continuously reminded them of their conditions [28]. This goes in line with our findings and it is possible that the insight from one of our respondents of tracking well-being rather than problems could make the work less burdensome. It could be one important strategy for finding the balance by reducing the experienced burden that focus on the illness creates. Sharon [29] has done work in self-tracking and found that there seems to be important considerations to make concerning autonomy, solidarity, and authenticity in relation to self-tracking as an element in personalised healthcare.

PwP in Sweden make the same conclusion as U.S. adults with other chronic conditions; that tracking affects the way they manage their health and communicate with healthcare providers, according to the knowledge they gain from tracking [30]. In a healthcare context, PD is often seen as mainly affecting movement whereas PwP often state that non-motor symptoms are at least equally bothersome. However, self-tracking and the right analysing tools could help also healthcare to see PD in a wider perspective. A previous study of perceptions of the use of technology for PD and epilepsy indicated that both healthcare professionals and PwP saw potential benefits for better understanding of the disease and improved disease management [14]. In that study, as in ours, PwP saw potential for improved selfcare, including medication management. Healthcare professionals in the previous study however saw risks with PwP adjusting their medications themselves [14]. Our study did not elicit the views of healthcare professionals but our respondents in both the interviews and the survey clearly expressed a desire to collaborate with healthcare and a frustration at the lack of effective tools. A large majority (82%) of our survey respondents expressed that healthcare needs to find better ways to assess PD on an individual level, stressing the importance of utilising tools such as self-tracking to achieve personalised healthcare. By ensuring that self-tracking is integrated in and accepted by healthcare the benefits of self-tracking could increase contributing to improving clinical management based on PwP selfcare. The findings of our study support the views of previous studies [28, 29] that the actual user perspective needs to be more in focus for self-tracking to truly support the much needed transformation of healthcare.

### Limitations

Our study is not without limitations. Our focus was on PwP in Sweden and the generalisability of our results is unclear. Seven interview respondents can be considered a small number. We were however less interested in representativity of all PwP and more interested in exploring the views and opinions of PwP with experience of self-tracking. We did reach saturation in the context of self-tracking, which indicates that the numbers were sufficient for the purpose of this mixed-method study. Another limitation concerns the survey. We chose to collect responses online which is a method generally suited for people who are already active and engaged. Furthermore, our PwP trackers survey respondents are relatively well educated, 62% have studied for more than 12 years. This can be compared to the general level of the Swedish population, which is 30–35% [31]. Above factors could mean that our results show more of what PwP who are already interested in self-tracking think and underestimates the problems involved. As well since one of the researchers (SR) has PD herself and has been conducting self-tracking for a long period of time, there could be a bias in underestimating the challenging aspects. We found however that risks and challenges were something that comes with long experience of tracking.

### Future research

Our study suggests that there is potentially a lot to be learned from PwP self-tracking on their own initiative and that the tools needed at least partly have distinctly different characteristics from tools used by and in healthcare. In this field, we have identified possible future work in the design and implementation of tools for measuring the “right” thing as well as for storing, analysing, visualising, and sharing data. We have also identified a number of other strategies that self-tracking patients apply to reduce the burden of tracking, e.g. focusing on tracking positive aspects rather than negative, or clearly limiting their tracking in both time and focus. It would be of interest to further explore how widely spread these strategies are and how effective they are in reducing the burden of self-tracking. We believe that the PDSA methodology could be a useful tool in exploring these issues further.

Another topic for further research is looking into the group that do not track. What can we learn from them? What are their reasons for not tracking?

We have also identified a neglected area in education related to self-tracking, both for PwP and healthcare professionals. With a better understanding of the needs for knowledge, both theoretical and practical, the benefits of self-tracking can be realised in a better way. Future work in this area include for example identifying appropriate methods and actors for education as well as organisational and funding issues.

Data from self-tracking efforts by individuals can also potentially be used for systematically improving healthcare and research, ultimately enabling personalised medicine. This would lead to a clearer focus on secondary prevention, which has the potential of improving health. This potential warrants further studies relating to, for example, how self-tracking could influence health economical aspects, both in healthcare as well as within the society.

## Conclusions

To the best of our knowledge, this is the first study exploring why and how PwP self-track and despite the limitations mentioned above, we believe that our results are an important contribution to extending the knowledge in the field of self-tracking in PD.

The extremely individual nature of PD makes it highly suited for self-tracking efforts and PwP who have tried think it entails both important benefits and burdens. The main identified benefits are that self-tracking gives PwP to a better understanding of their own specific manifestations of PD and contributes to a more effective decision making regarding their own selfcare. The process of self-tracking also enablesPwP to be more active in their communication with healthcare. This is important, especially considering the limited time PwP have with healthcare. We believe that this study’s main contribution is the insight that tracking takes a lot of work and there is a need to find the right balance between burdens and benefits. This balance can be as individual as the symptoms of PD itself, yet some strategies have been identified in this study; to focus on positive aspects rather than negative, to limit the focus of self-tracking both in time and scope, and to increase the benefits through improved tools and increased use of self-tracking results in the dialogue with healthcare. However, we still need a clearer understanding of these burdens and benefits, from the individual perspectives of every stakeholder, mainly PwP, healthcare professionals, and researchers respectively.

## Additional files

Additional file 1: Interview guide. (DOCX 15 kb)

Additional file 2: Survey questions. (DOCX 16 kb)

## Data Availability

Analyzed data for the study is available by the corresponding author.

## Abbreviations

DBS: Deep Brain Stimulation
PD: Parkinson Disease
PDSA: The Plan-Do-Study-Act cycle
PwP: People with Parkinson
