# Supplementary material for: “You have to know why you're doing this”: a mixed methods study of the benefits and burdens of self-tracking in Parkinson's disease
Source: BMC Med Inform Decis Mak. 2019 Aug 30;19:175. doi: 10.1186/s12911-019-0896-7 (PMC6716928; doi:10.1186/s12911-019-0896-7)
Supplement: Supplementary file 1 — Interview guide. (DOCX 15 kb) [file 12911_2019_896_MOESM1_ESM.docx]

# Interview guide self-tracking

Information about the study

- 1. OK to record?
  2. Time: no more than 1 hour
  3. Review the form for information and consent. Point out that participating in the interview is voluntary, they are free to not respond and free to interrupt the interview at any time.

Background information:

1. “Can you tell me a bit about yourself?”
   1. Age? Family situation? Where do they live? Profession?
   2. Disease history? Time of diagnosis? The situation around the diagnosis?
   3. Main symptoms? Which are most troublesome?
2. “Can you tell me about the healthcare you receive for your Parkinson’s disease?
   1. Frequency? Which aspects are you pleased with? Less pleased with?
   2. What does your current treatment consist of?
      1. Medications?
      2. Other treatments?
3. “Can you tell me about something that you do to improve your wellness that is not prescribed by healthcare?”

Self-tracking: Tell them what we mean by self-tracking, that we mean both things you measure using apps, devices and similar, but also observations made using pen and paper and even things you “just” track in your head.

1. Self-tracking:
   1. “Can you tell me what you know about self-tracking?”
   2. “Have you tried it yourself?/Are you interested in trying?”
   3. “Why are you interested/not interested?”
   4. “Which expectation/concerns do you have?”
2. Self-tracking and healthcare:
   1. “Do you have any thoughts around self-tracking and healthcare?” If they cant think of anything, nudge them by saying for example: “Do you think that healthcare would be interested in self-tracking?”
   2. “What are your thoughts around sharing self-tracking data with others, for example healthcare, other patients, family members?”
   3. “Do you see any risks/benefits/downsides?”
   4. “Can you describe what you would like the collaboration between you and healthcare around your Parkinson’s disease to look like?”
3. “Is there anything you would like to add?”
