# Supplementary material for: “You have to know why you're doing this”: a mixed methods study of the benefits and burdens of self-tracking in Parkinson's disease
Source: BMC Med Inform Decis Mak. 2019 Aug 30;19:175. doi: 10.1186/s12911-019-0896-7 (PMC6716928; doi:10.1186/s12911-019-0896-7)
Supplement: Supplementary file 2 — Survey questions. (DOCX 16 kb) [file 12911_2019_896_MOESM2_ESM.docx]

# Appendix 2 – Survey questions

A1. How long ago were you diagnosed with PD?

- Within the last year
- 1-5 years ago
- 6-10 years ago
- 11-15 years ago
- 16-20 years ago
- More than 20 years ago

A2. Are you… Male Female Prefer not to say

A3. Your age?

- Younger than 25
- 26-35 years
- 36-45 years
- 46-55 years
- 56-65 years
- 66-75 years
- 76-85 years
- 86 years or older

A4. Highest completed education?

- Compulsory school (<9 years)
- Upper secondary school (9-12 years)
- University (>12 years)

A5. County council of residence

B1. I use (or have in the past) the following methods to keep track of aspects relating to PD. (tick all that apply)

- Computer, smartphone (incl apps), tablet etc
- Sensor based technology, for example activity tracker, smartwatch etc
- Pen and paper
- I track in my head
- Other methods (please comment below)

B2. I would like to keep track of the following aspects relating to PD. (tick all that apply)

- Tremor
- Slowness of movement
- Rigidity/stiffness
- Walking difficulty/freezing of gait
- Mood/depression/anxiety
- Fatigue/sleepiness
- Gut issues/constipation/digestion
- Types of medication
- Timings of medication
- Medication side effects (for example dyskinesia)
- Diet
- Exercise
- Sleep
- Stress
- Other aspects of PD (please comment below)

B3. I keep track of (or have in the past) the following aspects relating to PD.

- Tremor
- Slowness of movement
- Rigidity/stiffness
- Walking difficulty/freezing of gait
- Mood/depression/anxiety
- Fatigue/sleepiness
- Gut issues/constipation/digestion
- Types of medication
- Timings of medication
- Medication side effects (for example dyskinesia)
- Diet
- Exercise
- Sleep
- Stress
- Other aspects of PD (please comment below)

B4. I keep track (or have in the past) of aspects relating to PD for the following reasons. Please indicate whether you agree or disagree with the following statements. (Options are: Strongly disagree, Disagree somewhat, Neither agree or disagree, Agree somewhat, Strongly agree)

- I enjoy tracking
- Tracking enables me to understand my PD better
- Tracking enables me to take an active approach in the management of my PD
- Tracking enables me to remember how my PD fluctuates over time
- I use tracking to prepare for healthcare visits
- I don't think tracking is useful for me
- Tracking helps me understand how my PD medications influence my symptoms and side effects
- Other reasons (please comment below)

B5. My approach to tracking of aspects relating to PD is (or has been in the past). Please indicate whether you agree or disagree with the following statements. (Options are: Strongly disagree, Disagree somewhat, Neither agree or disagree, Agree somewhat, Strongly agree)

- I try to track everything
- I try to track all the time
- I track sometimes
- I track specific things
- I think it is too difficult to track
- I rarely track my health
- I have had to learn a lot about PD to be able to benefit from tracking
- I find it difficult to know what to track
- I find it difficult to know how to track

B6. My use of tracking of aspects relating to PD is (or has been in the past). Please indicate whether you agree or disagree with the following statements. (Options are: Strongly disagree, Disagree somewhat, Neither agree or disagree, Agree somewhat, Strongly agree)

- I have made observations based on my tracking that have helped my understand me PD better
- I have changed things (for example medication type and/or timings, diet, exercise regimen etc) as a result of tracking
- I use tracking to decide if I need to contact healthcare professionals
- At some point in the past, I have been frustrated by how difficult it is to track different aspects of my health
- I share how I track my health with other people.
- I find it difficult to understand how to make sense of the things I track

B7. Tracking and healthcare. Please indicate whether you agree or disagree with the following statements. (Options are: Strongly disagree, Disagree somewhat, Neither agree or disagree, Agree somewhat, Strongly agree)

- I show my physician results from my tracking
- My physician is not interested in the results of my tracking
- My physician and I use results from my tracking in our discussions about my treatment
- My physician encourages my tracking
- I think healthcare should find better ways to evaluate/assess PD on an individual level

B8. Risks and challenges with tracking. Please indicate whether you agree or disagree with the following statements. (Options are: Strongly disagree, Disagree somewhat, Neither agree or disagree, Agree somewhat, Strongly agree)

- I think there is a risk for becoming obsessed with tracking PD
- I don't want tracking to get in the way of living
- I don't want to share my tracking data with healthcare
- I am open to sharing all my tracking data with anyone interested
- I don't want other people with PD to be able to see data of how my PD is progressing
- I think that you can track too much
